# Bone Marrow Aspiration Concentrate and Platelet Rich Plasma for Osteochondral Repair in a Porcine Osteochondral Defect Model

**DOI:** 10.1371/journal.pone.0071602

**Published:** 2013-08-12

**Authors:** Marcel Betsch, Johannes Schneppendahl, Simon Thuns, Monika Herten, Martin Sager, Pascal Jungbluth, Mohssen Hakimi, Michael Wild

**Affiliations:** 1 Department of Trauma and Hand Surgery, University Hospital Duesseldorf, Duesseldorf, Germany; 2 Clinic for Vascular and Endovascular Surgery, University Hospital Muenster, Muenster, Germany; 3 Central Animal Research Facility, University Hospital Duesseldorf, Duesseldorf, Germany; 4 Department of Trauma and Orthopaedic Surgery, Klinikum Darmstadt, Darmstadt, Germany; University of Minho, Portugal

## Abstract

**Background:**

Bone marrow aspiration concentrate (BMAC) may possess a high potency for cartilage and osseous defect healing because it contains stem cells and multiple growth factors. Alternatively, platelet rich plasma (PRP), which contains a cocktail of multiple growth factors released from enriched activated thrombocytes may potentially stimulate the mesenchymal stem cells (MSCs) in bone marrow to proliferate and differentiate.

**Methods:**

A critical size osteochondral defect (10×6 mm) in both medial femoral condyles was created in 14 Goettinger mini-pigs. All animals were randomized into the following four groups: biphasic scaffold alone (TRUFIT BGS, Smith & Nephew, USA), scaffold with PRP, scaffold with BMAC and scaffold in combination with BMAC and PRP. After 26 weeks all animals were euthanized and histological slides were cut, stained and evaluated using a histological score and immunohistochemistry.

**Results:**

The thrombocyte number was significantly increased (*p = 0.049*) in PRP compared to whole blood. In addition the concentration of the measured growth factors in PRP such as BMP-2, BMP-7, VEGF, TGF-β1 and PDGF were significantly increased when compared to whole blood (*p<0.05*). In the defects of the therapy groups areas of chondrogenic tissue were present, which stained blue with toluidine blue and positively for collagen type II. Adding BMAC or PRP in a biphasic scaffold led to a significant improvement of the histological score compared to the control group, but the combination of BMAC and PRP did not further enhance the histological score.

**Conclusions:**

The clinical application of BMAC or PRP in osteochondral defect healing is attractive because of their autologous origin and cost-effectiveness. Adding either PRP or BMAC to a biphasic scaffold led to a significantly better healing of osteochondral defects compared with the control group. However, the combination of both therapies did not further enhance healing.

## Introduction

Although autologous chondrocyte transplantation is a clinically successful strategy to treat chondral defects, the restoration of functional articular cartilage and subchondral bone is questionable [1]. Autologous osteochondral grafting is currently one of the few surgical options to address subchondral bone and articular cartilage at once [2]. However, autologous osteochondral grafts have limitations in size, a limited number of available implants and donor site morbidity, which may cause a future deterioration at the healthy donor site [3]. Additionally, the clinical outcomes of both techniques in prospective randomized trials are inconsistent [4], [5].

Using a resorbable biphasic scaffold for osteochondral repair would allow a single-step treatment of subchondral bone and articular cartilage. Furthermore, it would reduce donor-site morbidity, and permit long-term storage and on-demand use [6]. A combination of bone-marrow stimulation and resorbable scaffolds, which provide initial mechanical stability and allow a homogenous three-dimensional cell distribution, could be a promising treatment option [6] as indicated in multiple studies using a large variety of different scaffolds for osteochondral repair [7]–[9]. Mesenchymal stem cells (MSCs) are a promising cell type for osteochondral repair, because once obtained, e.g. from the iliac crest, they can differentiate into chondrocytes and osteoblasts [10]. However, in vitro expansion of MSCs holds various problems, e.g. sterility of the cell culture, high costs, use of fetal bovine serum and the length of the cultivation, which does require a time-delayed second operation [11]. A possible alternative could be the use of a perioperative stem cell concentrate, as a single-step procedure by means of density gradient centrifugation of autologous bone marrow [12], [13]. Cell-based therapy options using intraoperative, one-step procedures with progenitor cells from bone marrow have shown promising results in musculoskeletal tissues [13], [14]. Platelet rich plasma (PRP), which is defined as a fraction of autologous blood having a platelet concentration above baseline [15], contains a high concentration of growth factors with a positive effect on tissue healing and regeneration [16], [17]. Due of its autologous origin and low cost it has significant advantages over other therapies with recombinant growth factors. PRP extracted from autologous blood is less immunogenic and more biocompatible than recombinant growth factors, which often are descended from other species [18]. Furthermore, there is no risk of transmissible diseases and it can easily be obtained on the day of surgery from whole blood.

Since recent studies indicated that growth factors derived from platelets can stimulate the chondrogenic differentiation of bone marrow stem cells (BMSCs), enhance chondrocyte proliferation and extracellular matrix biosynthesis [19]–[22] combining both bone marrow aspiration concentrate (BMAC) and PRP to stimulate bone and chondrogenic healing in osteochondral defects may be effective. Therefore, the purpose of this present study was to evaluate the potential of PRP in combination with bone marrow aspiration concentrate BMAC in a scaffold for the treatment of an osteochondral defect in a large animal model.

## Materials and Methods

### Animals

Fourteen adult female Goettinger mini-pigs (aged 18–30 months, weight 25–35 kg) were used in this study. This study was carried out in strict accordance with the recommendations in the Guide for the Care and Use of Laboratory Animals of the National Institutes of Health. The local Animal Care and Use Committee of the Heinrich Heine University and local government of Duesseldorf (permit number: 87–51.04.2010.A140) approved the animal selection, management and the surgery protocol. All surgery was performed under sodium pentobarbital anaesthesia, and all efforts were made to minimize suffering.

### Bone Marrow Harvest and Concentration

Bone marrow (BM) aspirate was harvested from both iliac crests of 7 mini-pigs by Jamchidi vacuum aspiration. After aspiration the mononucleated cells were concentrated using a point-of-care device (MarrowStim® mini concentration system, Biomet Biologics, Inc., USA). One 30 ml syringe was filled with 6 ml of citrate anticoagulant (ACD-A, Anticoagulant Citrate Dextrose Solution, Biomet Biologics) and 2×12 ml of bone marrow. According to the manufacturers instructions this solution was centrifuged at 600×g for 15 min., then 3–4 ml of the nucleated cell concentrate (NCC) was separated from the cell free plasma/ACD-A mixture and from red blood cells. The concentration of the mononucleated cells in the original BM aspirate and in the separated NCC of the BMAC group and in the BMAC of the BMAC+PRP group prior to mixture was analysed with an automatic counter (ADVIAs 120®, Bayer Diagnostics GmbH, Germany). The concentration factor of the NCC was calculated from the quotient of mononuclear cells in BM/NCC of the BMAC and BMAC+PRP group.

### Platelet Rich Plasma (PRP) Preparation

PRP was prepared from 60 ml autologous whole blood, which was retrieved from the auricular vein of the mini-pigs directly prior to the surgery. The thrombocyte concentrate was produced using the “GPS II Platelet Separation System®” (Biomet Biologics, USA). Following the manufacturer’s protocol, to 6 ml of citrate anticoagulant 54 ml of autologous whole blood were added and then centrifuged at 3200 RPM for 15 minutes resulting in three different layers. The platelet-poor plasma (PPP) was then separated resulting in 10 ml of PRP. PRP was activated with autologous thrombin, which was produced from 7 ml of whole blood by centrifugation, and 2 ml of 10% calcium chloride. The thrombocyte count in whole blood and in the resulting PRP was analysed in an automatic cell counter. In addition, the concentration factor of PRP was calculated from the quotient of the thrombocyte count in whole blood and in PRP. Additionally, BMP-2, BMP-7, VEGF, TGF-β and PDGF were quantified in serum or plasma and in PRP using commercially available ELISA kits (Quantikine®, ELISA KITS, R&D Systems, USA).

### Animal Model and Surgery

**Figure 1:**
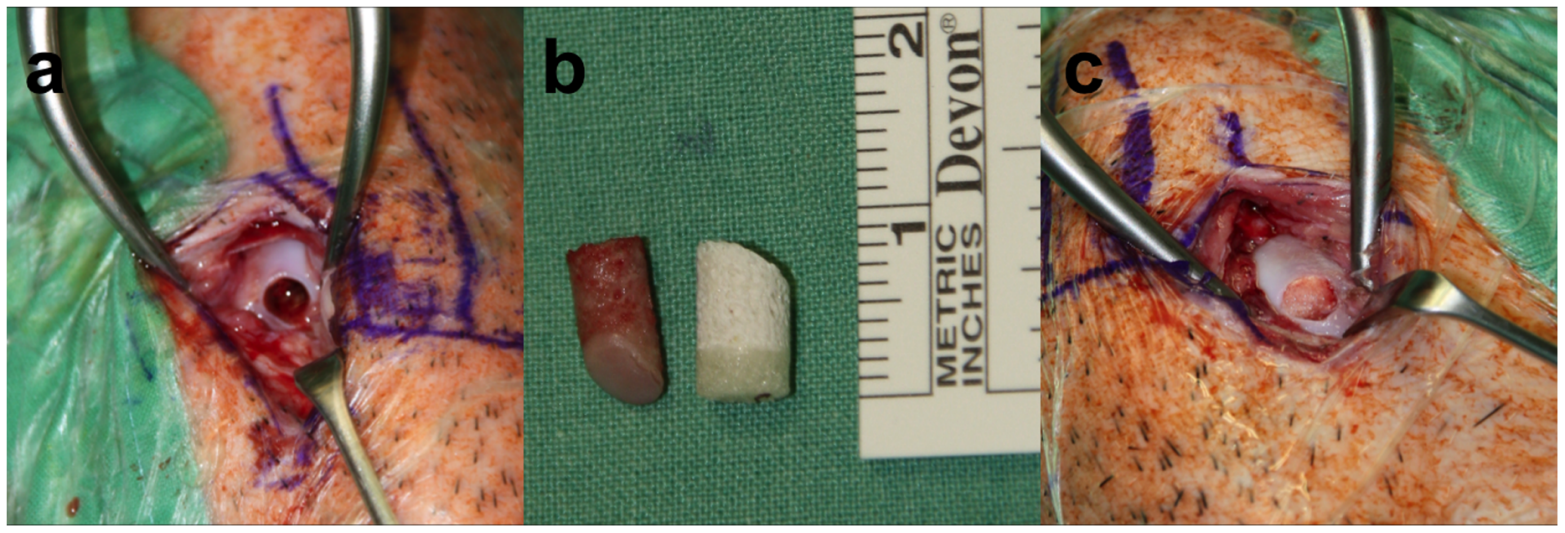
Osteochondral defect. In all animals, a 6×10 mm cylindrical osteochondral defect in the medial femoral condyles of both knee joints was surgically created with a cylindrical chisel (a). The osteochondral graft (left) from the defect was measured and then the biphasic scaffold (right) was cut to the respective length of the graft prior to implantation (b). The cut scaffold was then supplemented with the respective supplement and implanted into the condyle (c).

All mini-pigs were acclimated for one week before the surgery and were randomized, using a sealed envelope system, into the following four treatment groups: biphasic scaffold alone (control group), scaffold with PRP, scaffold with BMAC and scaffold with PRP and BMAC. Both knee joints of the 14 animals were used for the experiments resulting in a total number of 28 defects. In all animals, a 6×10 mm cylindrical osteochondral defect in the medial femoral condyle was surgically created with a cylindrical chisel. The osteochondral defect was filled in all groups with a biphasic scaffold (TRUFIT BGS^©^, Smith & Nephew, USA), which contains 50% non-crystalline amorphous poly-D,L-lactide-co-glycolide (PLGA), 10% poly(glycolic acid) fibres and 40% calcium sulfate (Figure 1). We chose this scaffold because previous studies have demonstrated promising results using this scaffold for osteochondral repair and because of its hydrophilic nature that facilitates absorption of PRP and BMAC [23], [24]. PRP, BMAC or the combination of PRP and BMAC were added to the scaffold prior to implantation. After activation of the PRP with autologous thrombin the scaffold was immersed in 2 ml of PRP for 5 minutes to ensure that PRP had been completely soaked up by the scaffold before polymerizing it to a platelet-rich gel. In the BMAC group, 2 ml of BMAC was added to the scaffold for 5 minutes, and in the PRP with BMAC group 1 ml BMAC and 1 ml PRP were mixed first and then after activation of the PRP, the scaffold was immersed for 5 minutes in this combined solution. In the control group the defects were filled with the biphasic scaffold alone.

One experienced surgeon performed all surgical procedures in aseptic conditions with the mini-pigs under general anaesthesia. The skin of both hind knee joints was scrubbed and disinfected. A midline skin incision was made and the medial femoral condyle was exposed through a medial parapatellar approach. A cylindrical osteochondral defect (diameter 6 mm, depth 10.0 mm) was made in the medial femoral condyle using a 6 mm mosaicplasty digging tool to ensure that the size of all defects was comparable. The biphasic osteochondral implant was press-fit into the defect until the scaffold was at the same level with the surface of the surrounding articular cartilage. Wound closure was accomplished with bioresorbable sutures, and after the procedure the animals were returned to the enclosure and monitored until fully recovered from the anaesthesia.

### Postoperative Management and Euthanasia

All mini-pigs were allowed to fully weight bear and to move freely without any constraints postoperatively. After 26 weeks the animals were euthanized with an overdose of 3% sodium pentobarbital (Eutha 77®, Essex Pharma GmbH, Germany). The knee joints were opened and macroscopically evaluated for signs of inflammation, such as tissue reddening, hypertrophy of the villous part of the synovial membrane, tissue adhesions and clarity and colour of the synovial fluid. The macroscopic appearance and quality of healing were assessed blindly using a scoring system. Thereafter, the medial femoral condyles of both knees were excised and fixed in 10% neutral buffered formalin solution for 14 days.

### Staining and Evaluating Specimens

The specimens were dehydrated using ascending grades of alcohol and xylene, then infiltrated, and embedded in methylmethacrylate for non-decalcified sectioning. Any negative influence of heat was avoided by performing controlled polymerization in a cold atmosphere (-4°C). Each specimen was cut in the sagittal direction using a diamond wire saw (Exakt®, Apparatebau, Norderstedt, Germany). Serial sections were prepared from the central parts of the defect areas, resulting in three sections each of approximately 320 µm thickness. All specimens were glued with acrylic cement (Technovit 7210 VLC®, Heraeus Kulzer) to oversize plastic slides (size: 50×100×2 mm, Dia-Plus®, Walter Messner GmbH, Germany), and ground to a final thickness of approximately 60 µm. The sections were stained with toluidine blue and safranin-o to evaluate the glycosaminoglycan (GAG) content and for histological assessment. Furthermore, a mouse monoclonal antibody reactive with porcine collagen II (1∶400, Acris Antibodies GmbH, Germany) and the corresponding nonspecific antibody (mouse IgG1) as a negative control were used to stain for collagen II in the sections. The macroscopic appearance of the defects was evaluated using a macroscopic scoring system introduced by Rudert et al [25]. For histological examination a modified O’Driscoll score (Table 1) was used [26]. Two independent experts double-blinded, who were familiar with the histology of cartilage repair, evaluated the defects. All scores were the means of the two independent evaluations.

**Table 1:** Modified O’Driscoll score.

	Score
**Nature of the predominant tissue**	
*Cellular morphology*	
Hyaline articular cartilage	4
Incompletely differentiated mesenchyme	2
Fibrous tissue	0
*Safranin-O staining of the matrix*	
Normal or nearly normal	3
Moderate	2
Slight	1
None	0
**Structural characteristics**	
*Surface regularity*	
Smooth and intact	3
Superficial horizontal lamination	2
Fissures: 25–100% of the thickness	1
Severe disruption, including fibrillation	0
*Structural integrity*	
Normal	2
Slight disruption, including cysts	1
Severe disintegration	0
*Thickness*	
100% of normal adjacent cartilage	2
50–100% of normal cartilage	1
<50% of normal cartilage	0
*Bonding to the adjacent cartilage*	
Bonded at both ends of graft	2
Bonded at one end, or partially at both ends	1
Not bonded	0
**Absence of cellular changes resulting from degeneration**	
*Hypocellularity*	
Normal cellularity	3
Slight hypocellularity	2
Moderate hypocellularity	1
Severe hypocellularity	0
*Chondrocyte clustering*	
No clusters	2
<25% of the cells	1
5–100% of the cells	0
*Absence of degenerative changes in adjacent cartilage*	
Normal cellularity, no clusters, normal staining	3
Normal cellularity, mild clusters, moderate staining	2
Mild or moderate hypocellularity, slight staining	1
Severe hypocellularity, poor or no staining	0
Total	24

To evaluate the quality of the BMAC and PRP, the following in vitro tests were performed.

### MSC Culture

The mononucleated cells from BM and from NCC were cultured in standard cell culture medium DMEM, with 1 g/l glucose, 20% FCS, 100 units/ml penicillin, 100 µg/ml streptomycin and 2 mM glutamine (PAA Laboratories GmbH, Austria). Culture conditions were 5% CO_2_ and 37°C.

### CFU Assay

The proliferation potency of the mononucleated cells was measured in colony-forming units (CFU). Equal concentrations of mononucleated cells from BM and from NCC were seeded in 24 - well plates at cell densities of 1×10^5^ cells/cm^2^. After 7 and 14 days of culture the cells were incubated for 60 min at room temperature with hematoxylin to determine the number of CFU-fibroblast-like cells (CFU-F).

### FACS Analysis

Due to the limited availability of commercial antibodies cross-reacting with the species pig, only the MSC markers CD14, CD44, CD90 and the hematopoietic markers CD45 and CD34 were measured by flow cytometry (Cytomics FC 500 FACS; Beckman Coulter, Germany) for phenotypic characterization of the cultivated cells.

### Lineage Differentiation and Corresponding Histological Staining

Adherent cells of the first passage were further cultivated for osteogenic, chondrogenic and adipogenic differentiation as described previously [27].

### Statistical Analysis

Unifactorial ANOVA was calculated to check for differences in the histological scores between the four groups. A t-Test was used to evaluate for differences in the growth factors. The level of significance was set at *p<0.05*. Statistical analysis and graphic presentation were prepared using software SPSS 20.0® (SPSS Inc., Chicago, USA).

## Results

### In vitro Analysis

**Figure 2:**
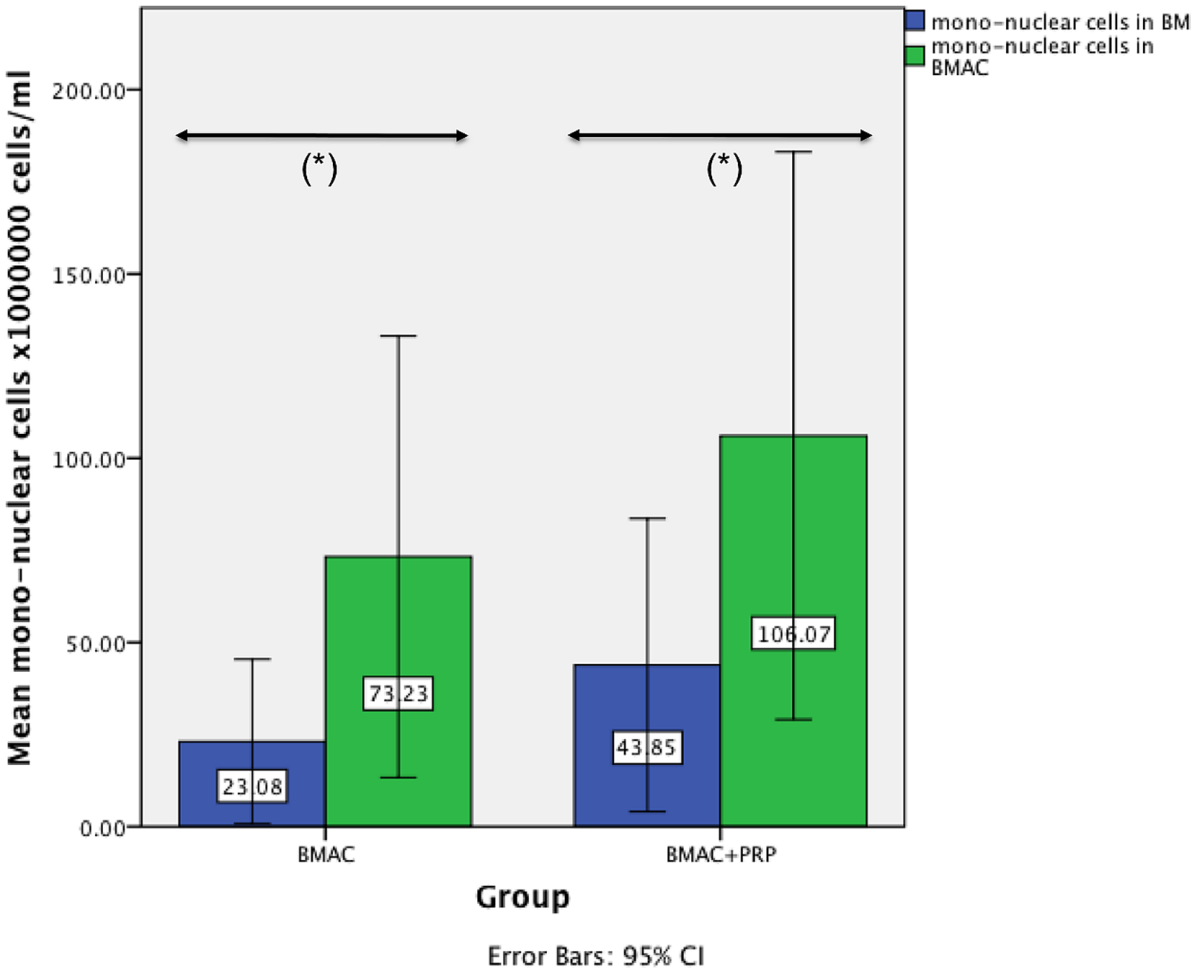
Mean mononuclear cell count. The mean mononuclear cell count of both treatment groups (BMAC and BMAC+PRP) in bone marrow (BM) and bone marrow aspiration concentrate (BMAC). In the BMAC group there was a 3.17 (*p = 0.021*) fold increase of mononuclear cells in BMAC compared to BM, and in the BMAC+PRP a 2.42 (*p = 0.006*) fold increase of the mononuclear cells.

**Figure 3:**
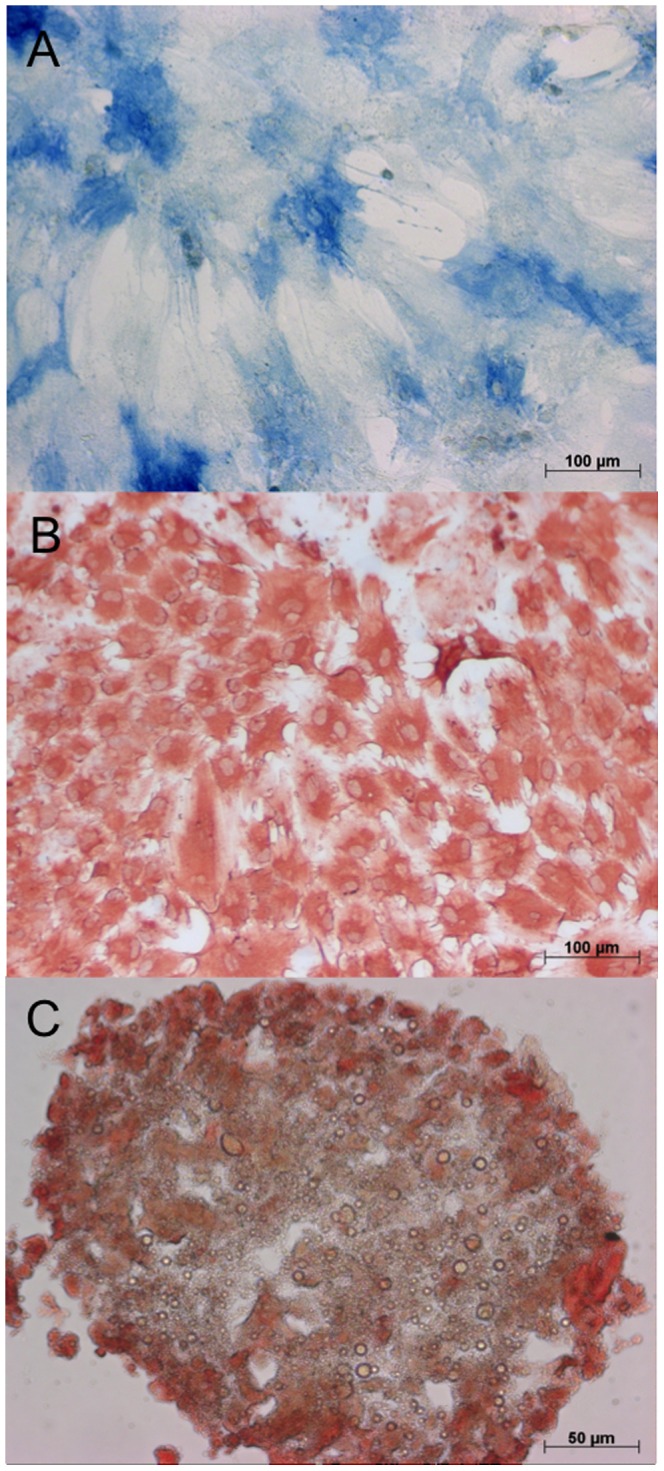
Differentiation assays on day 21. The differentiation of the mesenchymal stem cells from the bone marrow aspiration concentrate into osteoblasts became apparent by an intense blue staining for alkaline phosphatase activity (A). The adipocytes became apparent by the accumulation of lipid-rich vacuoles stained with PPAR (B) Cells cultured in chondrogenic medium showed a positive stain for chondrocyte markers over an increasing proportion of the pellet from 10 to 21 days (C).

**Figure 4:**
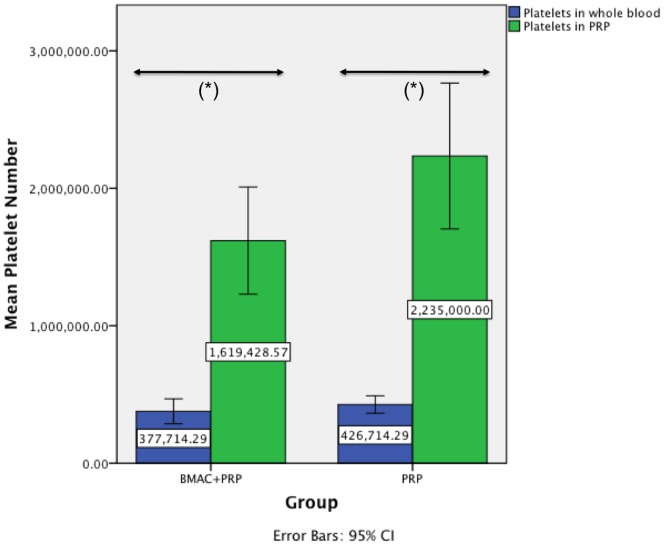
Mean number of platelets. The mean number of platelets in whole blood and in PRP in both treatment groups. In both groups we measured a significant increase (*p<0.001 and p = 0.049*) of the platelet number in PRP compared to whole blood.

The quality of the used BMAC was evaluated by a calculation of the concentration factor of the mononuclear cells in both BMAC groups compared to the mononuclear cells in bone marrow, the formation of colony forming units, a cell characterization using FACS analysis and the trilineage differentiation of the cells. The mean value of the mononuclear cells in the BM of the BMAC group was 23.08×10^6^ cells/ml ±24.12 and 43.85×10^6^ cells/ml ±43.00 in the PRP with BMAC group (Figure 2). In the BMAC group the mean concentration of mononuclear cells was 73.23×10^6^ cells/ml ±64.75 and 106.07×10^6^ cells/ml ±83.27 in the PRP with BMAC group. The resulting concentration factors of the mononuclear cells in BMAC was 3.17 (p = 0.021) in the BMAC only group and 2.42 (p = 0.006) in the PRP with BMAC group, a statistically significant higher level of mononuclear cells within the BMAC fraction compared with the BM in both groups. For all animals of the BMAC group, adherent cells reached confluence after 16–21 days. The number of CFU-F was counted in colonies consisting of more than 40 cells with a defined center. After 7 days the mean number of CFU-F in BM was 0.3±0.69 in 1×10^5^ cells/cm^2^, while in BMAC the mean CFU-F was 1.9±2.5 in 1×10^5^ cells/cm^2^ (*p = 0.144*). On day 14 the mean number of CFU-F in BM was 3.21±3.55 in 1×10^5^ cells/cm^2^, while in BMAC the mean CFU-F was 5.09±5.21 in 1×10^5^ cells/cm^2^ (*p = 0.456*). In BM there was no significant difference (*p = 0.078*) found between the mean CFU-F on day 7 and day 14. However, we found a significant difference of the CFU-F in BMAC between day 7 and 14 (*p = 0.035*). FACS analysis revealed positive staining for CD44 in more than 90% ±8.3 of all viable single cells. For CD14 91.2% ±7.6 of all viable single cells were stained positively, which is comparable to CD90 (89% ±11.3). The hematopoietic markers CD45 and CD34 could be detected on only 5.9% ±1.70 and 2.5% ±0.9 cells, respectively. The data demonstrate a predominant expression of cell surface molecules associated with MSC and a minimal expression of hematopoietic cell surface molecules. Cells could be successfully differentiated into osteogenic, chondrogenic and adipogenic cells in all animals. The differentiation of the cells into osteoblasts became apparent by an intense blue staining for alkaline phosphatase activity. Positive staining for the osteocalcin (red) was detected in more than 90% of all adherent cells as shown by counterstaining with hemalaun (Figure 3). The adipocytes became apparent by the accumulation of lipid-rich vacuoles. Cells cultured in chondrogenic medium showed a positive stain for chondrocyte markers over an increasing proportion of the pellet from 10 to 21 days. In the PRP group the platelet number was significantly increased (*p = 0.049*; 2.2±0.57×10^6^ cells) compared with the platelet number in whole blood (0.426±0.06×10^6^ cells), which was on average a 5.24-fold increase (Figure 4). In the PRP with BMAC group the platelet number was also significantly increased (*p<0.001*) in PRP (1.6±0.42×10^6^ cells) compared with the platelet number in whole blood (0.38±0.09×10^6^ cells), a 4.29-fold increase (Figure 4). The mean concentration of BMP-2 was 12.87±2.06 pg/ml in plasma and 167.7±83.42 pg/ml in PRP (*p = 0.001*), the mean concentration of BMP-7 was 95.85±37.28 pg/ml in plasma and 178.86±128.98 pg/ml in PRP (*p = 0.003*), the mean concentration of VEGF was 11.97±3.6 pg/ml in plasma and 77.35±3.6 pg/ml in PRP (*p = 0.001*), the mean concentration of TGF-β1 was 2899.71±855.50 pg/ml in plasma and 40904.84±5468.71 pg/ml in PRP (*p<0.001*) and the mean concentration of PDGF was 107.69±194.45 pg/ml in plasma and 13962.29±5127.81 pg/ml in PRP (*p<0.001*).

**Figure 5:**
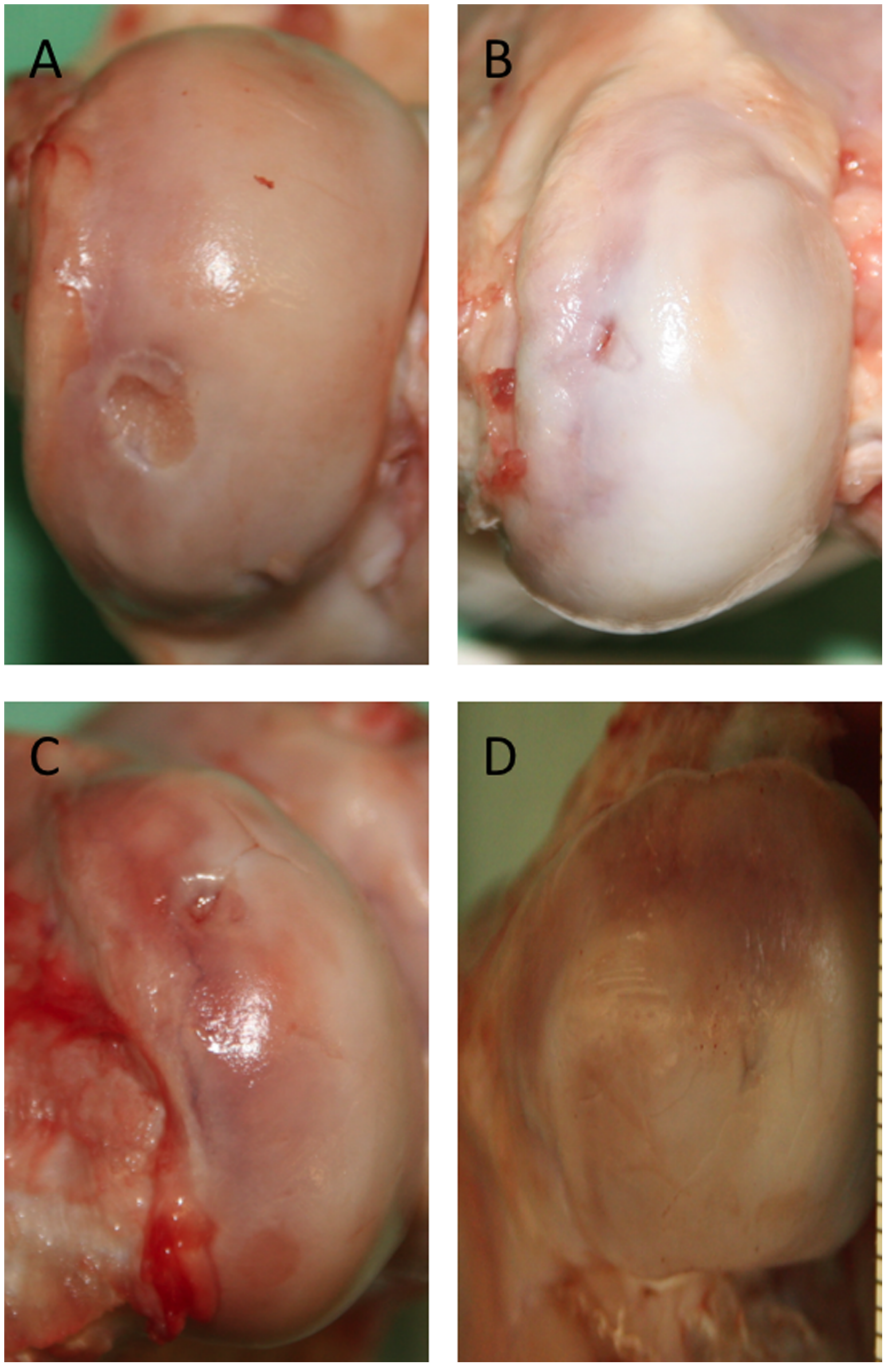
Representative macroscopic pictures of all four groups. In several cases a central depression was noted in the group, where the TRUFIT plug was implanted without any further supplement (A). In some cases, smaller residues of the defect were still visible in the PRP (B), BMAC (C) or BMAC+PRP (D) treatment groups.

All animals were ambulatory after the surgery, indicating the feasibility of using a bilateral defect model. Gross examination of the knee joints revealed that there were no abrasions on the opposing articulating surfaces, and no inflammation of the synovial membrane or other joint tissues were noted in any defects. None of the implanted scaffolds appeared dislodged. In no defect was the biphasic scaffold sitting above the native cartilage (Figure 5). Macroscopic examination of the explanted knee joints showed no lesions on the corresponding articular surfaces, and no inflammation of the synovial membrane was recognized. None of the 28 implanted scaffolds was dislodged. We found a mean macroscopic score for the control group of 5.57±1.62, of 5.86±1.77 for the PRP group, 6.57±1.13 for the BMAC group and 5.86±1.35 for the BMAC+PRP group. No significant differences (p>0.05) were found between the groups for the macroscopic score.

### Histology

**Figure 6:**
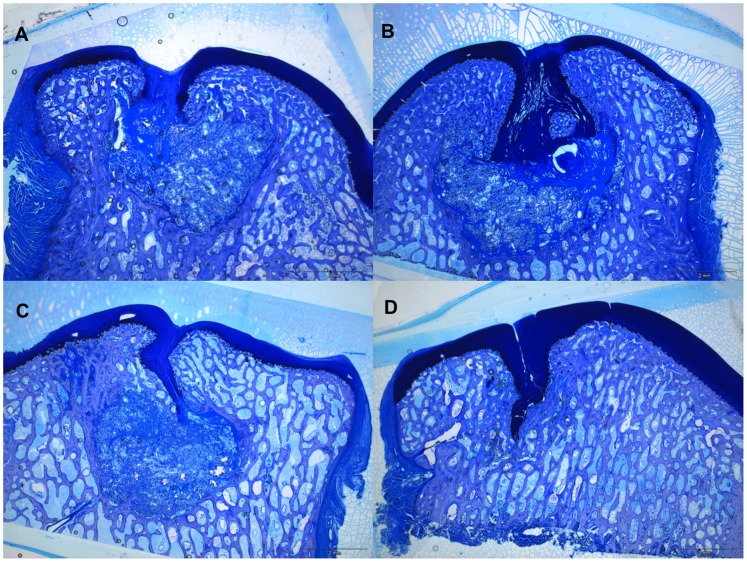
Representative histological slides. Representative histological slides (stained with toluidine-blue) of each of the investigated groups, a) scaffold only b) PRP c) BMAC d) BMAC with PRP. In the treatment groups the regenerated tissue stained positive for sGAGs.

**Figure 7:**
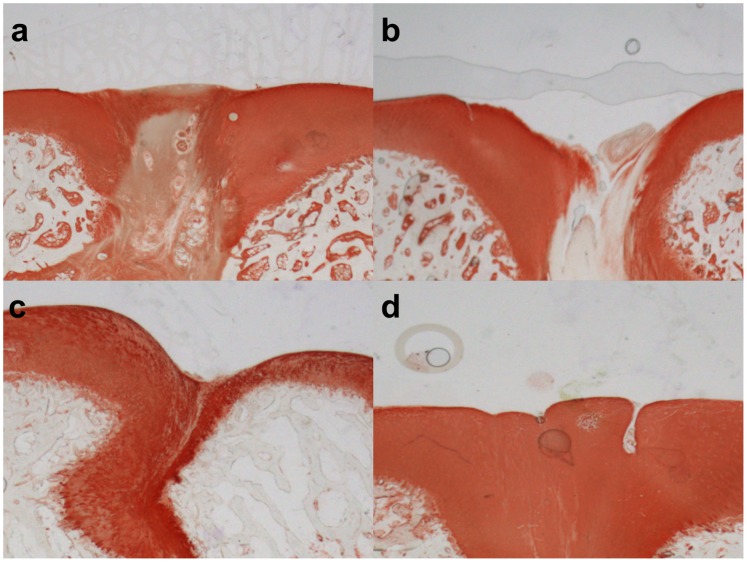
Immunohistochemical analysis for collagen II. Immunohistochemical analysis for collagen II of the regenerate cartilage in representative slides of all four groups. a) scaffold only b) PRP c) BMAC d) BMAC with PRP. No signs of degenerative changes in the adjacent cartilage were found. In the defects of the therapy groups, areas of chondrogenic tissue, which contained collagen II on the basis of positive immunostaining, were present. However, in the control group the regenerative tissue was generally fibrous. This tissue was deficient in collagen type II as shown by specific staining.

**Figure 8:**
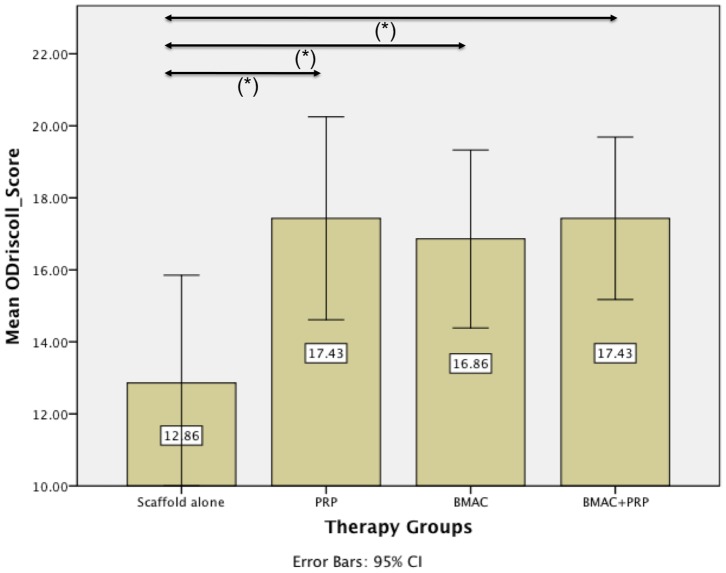
Results modified O’Driscoll histological scoring. The lowest score in the histological score (12.86±3.24) was found for the biphasic scaffold alone (control group). Adding bone marrow aspiration concentrate (16.86±2.67) or platelet rich plasma (17.43±3.05) to the scaffold led to a significant increase in the histological score (*p<0.05*). However, the combination of BMAC and PRP with the biphasic scaffold (17.43±2.44) did not further enhance the histological score.

The newly formed tissue in the cartilage area of the defects in the therapy groups stained blue with toluidine blue and contained collagen II on the basis of positive immunostaining (Figure 6 and Figure 7). In contrast, mostly fibrous tissue was found in defects of the control group, which did not stain for collagen type II or sulphated glycosaminoglycans (sGAG). Remnants of the implanted scaffolds were consistently present in the osseous phase of the defects, while the cartilage phase was completely replaced after 26 weeks. The repair tissue in the bony phase was fibrous with vascularisation and giant cells, indicating an on-going degradation process of the scaffold at 26 weeks. In ten of the twenty-eight defects a subchondral cyst was noted in the subchondral layer. Furthermore, the normal architecture of the cancellous bone has not been restored after 26 weeks. The lowest score in the modified O’Driscoll score (12.86±3.24) was found for the biphasic scaffold alone (control group) (Figure 8). Adding bone marrow aspiration concentrate (16.86±2.67) or platelet rich plasma (17.43±3.05) to the scaffold led to a significant increase in the score (*p<0.05*) compared with the control group. The combination of BMAC and PRP with the biphasic scaffold (17.43±2.44) also led to a significant increase of the score (*p<0.05*) as compared to the control group, but it did not further enhance the histological score when compared to the BMAC and PRP group (Figure 8).

## Discussion

Articular cartilage has a limited capacity for self-repair because of the low mitotic activity of chondrocytes and the avascularity of hyaline cartilage. In weight bearing joints large articular cartilage defects fail to heal spontaneously and may often result in progressive deterioration and osteoarthritis. The goal of current treatments is still only to prevent or delay the development of osteoarthritis [28]. The results of this study show that the combination of BMAC or PRP in a biphasic scaffold can significantly improve osteochondral healing in mini-pigs. However, the combination of both BMAC and PRP did not further enhance the histological score. We used a bilateral defect model in the medial femoral condyles of mini-pigs with a diameter of 6 mm and a depth of 10 mm to evaluate the influence of BMAC and PRP on the osteochondral defect healing. Following Jung et al. [29] defects of 10 mm depth and 5.4 mm diameter are considered a critical osteochondral defect in this species. We chose the mini-pig as an animal model because its knee joint resembles the human knee joint dimensionally, and it also has a 1.5–2.0 mm thick cartilage surface, which makes it useful for the evaluation of cartilage repair [30], [31]. The poly lactic-glycolic acid (PLGA) biphasic scaffold used in our study is commercially available in Europe and approved for use in osteochondral defects. This scaffold is a multiphasic implant, composed of polyglycolic acid and poly-D,L-lactide–co-glycolide fibers preferentially aligned to provide a structural scaffold with pores, which provide space to allow cellular ingrowth. Calcium sulfate forms 10% of the plug, and there are also trace amounts of surfactant within the scaffold. This bilayer design provides cartilage and bone phases yielding comparable mechanical properties to the host tissue. In the cartilage phase, the material is softer and malleable enough to be physically contoured to the joint surface. The results of this study show that the newly formed tissue integrated well with the native cartilage. The implant is designed to be completely resorbed over time, allowing for a complete filling of the defect with repair tissue [32]. However, in our study and in a study by Joshi et al. [33] remnants of the scaffold in the bony phase were still present, possibly indicating its failure to restore the subchondral bone. These findings are in accordance with the work of Jiang et al. [34] who observed a substantial amount of a DL-poly-lactide-co-glycolide and b-tricalcium phosphate biphasic scaffold remaining in the osseous phase after 26 weeks, while the chondral phase of the implant was completely replaced by regenerative tissue. The newly formed tissue in the bony phase had an immature and disorganized structure with an infiltration of mononuclear and plasmatic cells. In ten of the twenty-eight defects, a cyst was found in the subchondral bone at the site of the scaffold. This could indicate that even after 26 weeks the cancellous bone adjacent to the implanted material is still undergoing a remodeling process and that the remnants of the scaffold itself could have impaired the histological results. Getgood et al. in their osteochondral defect study noted subchondral bone cysts in groups treated with a PLGA scaffold and suggested this as part of the healing pattern in ostechondral defects in the early phase of regeneration at 26 weeks [35]. The degradation of PLGA is caused by hydrolytic cleavage of ester bonds, which releases lactic and glycolic acid into the microenvironment within the material [36]. Getgood et al. [35] suggested that the decrease in pH may have a detrimental effect on cells and acid-sensitive calcium-phosphate precipitates in the osseous compartment, which could cause subchondral bone cysts during the course of scaffold resorption.

Platelet rich plasma (PRP) is defined as a fraction of autologous blood having a platelet concentration above baseline [15]. It is a rich source of autologous growth factors, such as PDGF, TGF- β, bFGF, IGF, VEGF, epithelial cell growth factor (ECGF) [2], as well as other factors [37] and can therefore be used as an alternative option to recombinant growth factors. PRP can be easily obtained on the day of surgery from autologous whole blood without a risk of disease transmission or immunogenic reaction. Multiple in vitro studies have shown a positive effect of PRP on the maintenance of the chondrocyte phenotype during expansion [38] as well as on chondrocyte proliferation and matrix production [19], [39]. Positive effects of PRP on cartilage restoration and glycosaminoglycan content were also shown in a rabbit chondral defect study using PRP in addition to a bilayer scaffold [40]. In a study by Milano et al. [41] the combination of microfracture with PRP showed a significant improvement in cartilage repair. Furthermore, Sun et al. [42] demonstrated in their osteochondral defect rabbit study that defects treated with a combination of PRP in a PLGA scaffold healed significantly better than the scaffold alone. PRP preparation is not standardized due to the use of multiple centrifugation protocols, different quantities of blood harvested and different quantities of PRP obtained, which all can result in a variability of PRP performance and growth factor concentration [43], [44]. Kon et al. e.g. found negative effects of growth factors from platelet rich plasma, when used in combination with a hydroxyapatite-collagen scaffold in sheep [45]. However, they did not report the number of platelets in the PRP that was used in their study, which is important since platelet concentrations in PRP between three to five times higher than in whole blood seem to be advantageous in tissue healing and higher or lower concentrations seem to have adverse effects [46], [47]. The point-of-care device that was used in our study led to a platelet concentration between 4.3 to 5.2 fold in PRP compared to whole blood indicating a beneficial concentration of the platelets in PRP. In addition the concentration of the measured growth factors in PRP such as BMP-2, BMP-7, VEGF, TGF-β1 and PDGF were significantly increased when compared with whole blood. The analysis of the PRP used in this study indicates that it was sufficiently replete with concentrated growth factors to allow the investigation of its effects on osteochondral healing. In future studies we also recommend that the number of leukocytes in PRP should be determined since studies have shown a potential influence of elevated numbers of leukocytes in PRP on the clinical outcome [48], [49].

Recent studies demonstrated that growth factors derived by platelets could stimulate chondrogenic differentiation of bone marrow derived mesenchymal stem cells, enhance chondrocyte proliferation and extracellular matrix biosynthesis [19]–[22], [50]–[52]. BMAC has been shown to be a good source for MSCs and growth factors in the regeneration of cartilage and hard tissues [53], [54]. CFU-F counts were used as a quality marker for MSCs since they indicate the number of transplanted progenitor cells [55]. In our study the number of CFU-F in BMAC (5.09±5.21 in 1×105 cells/cm^2^) was significantly higher than BM. These results are in accordance with the findings of other studies where mean values between 4.1 and 5.5 CFU-F/cm^2^ were measured in BMAC [13], [56]. The higher number of CFU-F from BMAC compared with BM is presumably due to the higher amount of mesenchymal progenitor cells and the reduction in the number of other cell types in the BMAC, created by the gradient centrifugation. The concentration factor of mononuclear cells in both BMAC groups was between 2.42 and 3.17, which was a significant increase compared to BM for both groups. Jäger et al. and Hermann et al. had a 5.2 and 4.4 fold increase in mononuclear cells in human BMAC compared to BM using the same concentration device, respectively [13], [57]. The lower number of mononuclear cells in our study may be due to species differences in cell size and density between humans and pigs. We were able to demonstrate the quality of the MSCs in BMAC by chondro-, osteo- and adipogenesis of the MSCs and positive staining for mesenchymal stem cell surface markers using FACS analysis.

The kinetics of cartilage and bone regrowth exceeds a 6-month evaluation period. Therefore, it must be considered that the osteochondral repair is probably still in evolution at the time of the evaluation in our study. However, we believe that a 6-month follow-up is sufficient to provide an indication of the ongoing regenerative process. A limitation of our study is that we evaluated the osteochondral repair in an acute, but not chronic mini-pig model. This has to be kept in mind when interpreting the results of our study, especially since in humans most of the defects that need treatment are of chronic nature. The addition of BMAC or PRP and the combination of BMAC with PRP in a biphasic scaffold led to a significant improvement of the osteochondral defect healing compared with the biphasic scaffold alone. Despite this significant improvement in the histological score, the main repair tissue in the cartilage layer was mostly fibrocartilage. Interestingly, the combination of BMAC with PRP did not further enhance the histological score when compared to the BMAC or PRP group. An explanation for this could be that both BMAC and PRP contain high levels of growth factors and in PRP it was demonstrated that too high levels of growth factors could have adverse effects on tissue healing [58]. A further explanation could be that we used a combination of 1 ml of BMAC and 1 ml of PRP in the BMAC+PRP group, instead of 2 ml in the respective BMAC and PRP alone groups, which would result in a lower total cellularity in the BMAC+PRP group.

### Conclusions

The biphasic scaffold used in this study led to mostly fibrous tissue in the defects of the control group. However, adding either PRP or BMAC resulted in a significantly better healing of the osteochondral defect compared with the control group. The clinical application of BMAC or PRP is attractive because of their autologous origin and cost-effectiveness, but the combination of both therapies did not further enhance healing in our study. In future studies different scaffolds should be evaluated as alternatives to the TRUFIT implant since the scaffold-only group had not produced a substantial repair.

## References

[1] Gomoll AH, Madry H, Knutsen G, van Dijk N, Seil R, et al (2010) The subchondral bone in articular cartilage repair: current problems in the surgical management. Knee Surg Sports Traumatol Arthrosc 18: 434–447. 20130833 10.1007/s00167-010-1072-x PMC2839476

[2] Gotterbarm T, Richter W, Jung M, Berardi Vilei S, Mainil-Varlet P, et al (2006) An in vivo study of a growth-factor enhanced, cell free, two-layered collagen-tricalcium phosphate in deep osteochondral defects. Biomaterials 27: 3387–3395. 16488472 10.1016/j.biomaterials.2006.01.041

[3] Smith GD, Knutsen G, Richardson JB (2005) A clinical review of cartilage repair techniques. J Bone Joint Surg Br 87: 445–449. 15795189 10.1302/0301-620X.87B4.15971

[4] Bentley G, Biant LC, Carrington RW, Akmal M, Goldberg A, et al (2003) A prospective, randomised comparison of autologous chondrocyte implantation versus mosaicplasty for osteochondral defects in the knee. J Bone Joint Surg Br 85: 223–230. 12678357 10.1302/0301-620x.85b2.13543

[5] Horas U, Pelinkovic D, Herr G, Aigner T, Schnettler R (2003) Autologous chondrocyte implantation and osteochondral cylinder transplantation in cartilage repair of the knee joint. A prospective, comparative trial. J Bone Joint Surg Am 85-A: 185–192. 12571292 10.2106/00004623-200302000-00001

[6] Siclari A, Mascaro G, Gentili C, Cancedda R, Boux E (2012) A cell-free scaffold-based cartilage repair provides improved function hyaline-like repair at one year. Clin Orthop Relat Res 470: 910–919. 21965060 10.1007/s11999-011-2107-4 PMC3270167

[7] Niederauer GG, Slivka MA, Leatherbury NC, Korvick DL, Harroff HH, et al (2000) Evaluation of multiphase implants for repair of focal osteochondral defects in goats. Biomaterials 21: 2561–2574. 11071606 10.1016/s0142-9612(00)00124-1

[8] Schaefer D, Martin I, Jundt G, Seidel J, Heberer M, et al (2002) Tissue-engineered composites for the repair of large osteochondral defects. Arthritis Rheum 46: 2524–2534. 12355501 10.1002/art.10493

[9] Tanaka T, Komaki H, Chazono M, Fujii K (2005) Use of a biphasic graft constructed with chondrocytes overlying a beta-tricalcium phosphate block in the treatment of rabbit osteochondral defects. Tissue Eng 11: 331–339. 15738686 10.1089/ten.2005.11.331

[10] Yoo JU, Barthel TS, Nishimura K, Solchaga L, Caplan AI, et al (1998) The chondrogenic potential of human bone-marrow-derived mesenchymal progenitor cells. J Bone Joint Surg Am 80: 1745–1757. 9875932 10.2106/00004623-199812000-00004

[11] Gan Y, Dai K, Zhang P, Tang T, Zhu Z, et al (2008) The clinical use of enriched bone marrow stem cells combined with porous beta-tricalcium phosphate in posterior spinal fusion. Biomaterials 29: 3973–3982. 18639333 10.1016/j.biomaterials.2008.06.026

[12] Hatzokos I, Stavridis SI, Iosifidou E, Karataglis D (2011) Christodoulou (2011) A Autologous bone marrow grafting combined with demineralized bone matrix improves consolidation of docking site after distraction osteogenesis. J Bone Joint Surg Am 93: 671–678. 21471421 10.2106/JBJS.J.00514

[13] Jager M, Herten M, Fochtmann U, Fischer J, Hernigou P, et al (2011) Bridging the gap: bone marrow aspiration concentrate reduces autologous bone grafting in osseous defects. J Orthop Res 29: 173–180. 20740672 10.1002/jor.21230

[14] Hendrich C, Franz E, Waertel G, Krebs R, Jager M (2009) Safety of autologous bone marrow aspiration concentrate transplantation: initial experiences in 101 patients. Orthop Rev (Pavia) 1: e32. 21808691 10.4081/or.2009.e32 PMC3143993

[15] Marx RE (2001) Platelet-rich plasma (PRP): what is PRP and what is not PRP? Implant Dent 10: 225–228. 11813662 10.1097/00008505-200110000-00002

[16] Alsousou J, Thompson M, Hulley P, Noble A, Willett K (2009) The biology of platelet-rich plasma and its application in trauma and orthopaedic surgery: a review of the literature. J Bone Joint Surg Br 91: 987–996. 19651823 10.1302/0301-620X.91B8.22546

[17] Eppley BL, Woodell JE, Higgins J (2004) Platelet quantification and growth factor analysis from platelet-rich plasma: implications for wound healing. Plast Reconstr Surg 114: 1502–1508. 15509939 10.1097/01.prs.0000138251.07040.51

[18] Wei LC, Gao SG, Xu M, Jiang W, Tian J, et al (2012) A novel hypothesis: the application of platelet-rich plasma can promote the clinical healing of white-white meniscal tears. Med Sci Monit 18: 47–50. 10.12659/MSM.883254 PMC3560705 22847210

[19] Akeda K, An HS, Okuma M, Attawia M, Miyamoto K, et al (2006) Platelet-rich plasma stimulates porcine articular chondrocyte proliferation and matrix biosynthesis. Osteoarthritis Cartilage 14: 1272–1280. 16820306 10.1016/j.joca.2006.05.008

[20] Drengk A, Zapf A, Sturmer EK, Sturmer KM, Frosch KH (2009) Influence of platelet-rich plasma on chondrogenic differentiation and proliferation of chondrocytes and mesenchymal stem cells. Cells Tissues Organs 189: 317–326. 18689989 10.1159/000151290

[21] Johnstone B, Hering TM, Caplan AI, Goldberg VM, Yoo JU (1998) In vitro chondrogenesis of bone marrow-derived mesenchymal progenitor cells. Exp Cell Res 238: 265–272. 9457080 10.1006/excr.1997.3858

[22] Mishra A, Tummala P, King A, Lee B, Kraus M, et al (2009) Buffered platelet-rich plasma enhances mesenchymal stem cell proliferation and chondrogenic differentiation. Tissue Eng Part C Methods 15: 431–435. 19216642 10.1089/ten.tec.2008.0534 PMC2819709

[23] Hindle P, Hendry JL, Keating JF, Biant LC (2013) Autologous osteochondral mosaicplasty or TruFit plugs for cartilage repair. Knee Surg Sports Traumatol Arthrosc. 10.1007/s00167-013-2493-0 23589126

[24] Bekkers JE, Bartels LW, Vincken KL, Dhert WJ, Creemers LB, et al.. (2013) Articular Cartilage Evaluation After TruFit Plug Implantation Analyzed by Delayed Gadolinium-Enhanced MRI of Cartilage (dGEMRIC). Am J Sports Med. 10.1177/0363546513483536 23585485

[25] Rudert M, Wilms U, Hoberg M, Wirth CJ (2005) Cell-based treatment of osteochondral defects in the rabbit knee with natural and synthetic matrices: cellular seeding determines the outcome. Arch Orthop Trauma Surg 125: 598–608. 16075272 10.1007/s00402-005-0008-2

[26] Rudert M (2002) Histological evaluation of osteochondral defects: consideration of animal models with emphasis on the rabbit, experimental setup, follow-up and applied methods. Cells Tissues Organs 171: 229–240. 12169820 10.1159/000063125

[27] Herten M, Sager M, Benga L, Fischer CJ, Jaeger M, et al (2013) Bone marrow concentrate for autologous transplantation in minipigs; characterization and osteogenic potential of MSC. Vet Comp Orthop Traumatol 26: 34–41. 23171924 10.3415/VCOT-11-11-0165

[28] Buckwalter JA, Mankin HJ (1998) Articular cartilage: degeneration and osteoarthritis, repair, regeneration, and transplantation. Instr Course Lect 47: 487–504. 9571450

[29] Jung M, Breusch S, Daecke W, Gotterbarm T (2009) The effect of defect localization on spontaneous repair of osteochondral defects in a Gottingen minipig model: a retrospective analysis of the medial patellar groove versus the medial femoral condyle. Lab Anim 43: 191–197. 19116289 10.1258/la.2008.007149

[30] Ahern BJ, Parvizi J, Boston R, Schaer TP (2009) Preclinical animal models in single site cartilage defect testing: a systematic review. Osteoarthritis Cartilage 17: 705–713. 19101179 10.1016/j.joca.2008.11.008

[31] Chu CR, Szczodry M, Bruno S (2010) Animal models for cartilage regeneration and repair. Tissue Eng Part B Rev 16: 105–115. 19831641 10.1089/ten.teb.2009.0452 PMC3121784

[32] Slivka MA, Leatherbury NC, Kieswetter K, Niederauer GG (2001) Porous, resorbable, fiber-reinforced scaffolds tailored for articular cartilage repair. Tissue Eng 7: 767–780. 11749733 10.1089/107632701753337717

[33] Joshi N, Reverte-Vinaixa M, Diaz-Ferreiro EW, Dominguez-Oronoz R (2012) Synthetic resorbable scaffolds for the treatment of isolated patellofemoral cartilage defects in young patients: magnetic resonance imaging and clinical evaluation. Am J Sports Med 40: 1289–1295. 22491793 10.1177/0363546512441585

[34] Jiang CC, Chiang H, Liao CJ, Lin YJ, Kuo TF, et al (2007) Repair of porcine articular cartilage defect with a biphasic osteochondral composite. J Orthop Res 25: 1277–1290. 17576624 10.1002/jor.20442

[35] Getgood AM, Kew SJ, Brooks R, Aberman H, Simon T, et al (2011) Evaluation of early-stage osteochondral defect repair using a biphasic scaffold based on a collagen-glycosaminoglycan biopolymer in a caprine model. Knee 19: 422–430. 21620711 10.1016/j.knee.2011.03.011

[36] Antheunis H, van der Meer JC, de Geus M, Heise A, Koning CE (2010) Autocatalytic equation describing the change in molecular weight during hydrolytic degradation of aliphatic polyesters. Biomacromolecules 11: 1118–1124. 20187614 10.1021/bm100125b

[37] Ham O, Song BW, Lee SY, Choi E, Cha MJ, et al (2012) The role of microRNA-23b in the differentiation of MSC into chondrocyte by targeting protein kinase A signaling. Biomaterials 33: 4500–4507. 22449550 10.1016/j.biomaterials.2012.03.025

[38] Spreafico A, Chellini F, Frediani B, Bernardini G, Niccolini S, et al (2009) Biochemical investigation of the effects of human platelet releasates on human articular chondrocytes. J Cell Biochem 108: 1153–1165. 19731249 10.1002/jcb.22344

[39] Ishida K, Kuroda R, Miwa M, Tabata Y, Hokugo A, et al (2007) The regenerative effects of platelet-rich plasma on meniscal cells in vitro and its in vivo application with biodegradable gelatin hydrogel. Tissue Eng 13: 1103–1112. 17348798 10.1089/ten.2006.0193

[40] Qi YY, Chen X, Jiang YZ, Cai HX, Wang LL, et al (2009) Local delivery of autologous platelet in collagen matrix simulated in situ articular cartilage repair. Cell Transplant 18: 1161–1169. 19660173 10.3727/096368909X12483162197169

[41] Milano G, Sanna Passino E, Deriu L, Careddu G, Manunta L, et al (2010) The effect of platelet rich plasma combined with microfractures on the treatment of chondral defects: an experimental study in a sheep model. Osteoarthritis Cartilage 18: 971–980. 20433936 10.1016/j.joca.2010.03.013

[42] Sun Y, Feng Y, Zhang CQ, Chen SB, Cheng XG (2009) The regenerative effect of platelet-rich plasma on healing in large osteochondral defects. Int Orthop 34: 589–597. 19434411 10.1007/s00264-009-0793-2 PMC2903145

[43] Borzini P, Mazzucco L (2005) Tissue regeneration and in loco administration of platelet derivatives: clinical outcome, heterogeneous products, and heterogeneity of the effector mechanisms. Transfusion 45: 1759–1767. 16271101 10.1111/j.1537-2995.2005.00600.x

[44] Weibrich G, Kleis WK, Hitzler WE, Hafner G (2005) Comparison of the platelet concentrate collection system with the plasma-rich-in-growth-factors kit to produce platelet-rich plasma: a technical report. Int J Oral Maxillofac Implants 20: 118–123. 15747683

[45] Kon E, Filardo G, Delcogliano M, Fini M, Salamanna F, et al (2010) Platelet autologous growth factors decrease the osteochondral regeneration capability of a collagen-hydroxyapatite scaffold in a sheep model. BMC Musculoskelet Disord 11: 220. 20875101 10.1186/1471-2474-11-220 PMC2954989

[46] Pieri F, Lucarelli E, Corinaldesi G, Fini M, Aldini NN, et al (2009) Effect of mesenchymal stem cells and platelet-rich plasma on the healing of standardized bone defects in the alveolar ridge: a comparative histomorphometric study in minipigs. J Oral Maxillofac Surg 67: 265–272. 19138598 10.1016/j.joms.2008.06.036

[47] Weibrich G, Hansen T, Kleis W, Buch R, Hitzler WE (2004) Effect of platelet concentration in platelet-rich plasma on peri-implant bone regeneration. Bone 34: 665–671. 15050897 10.1016/j.bone.2003.12.010

[48] Castillo TN, Pouliot MA, Kim HJ, Dragoo JL (2011) Comparison of growth factor and platelet concentration from commercial platelet-rich plasma separation systems. Am J Sports Med 39: 266–271. 21051428 10.1177/0363546510387517

[49] McCarrel TM, Minas T, Fortier LA (2012) Optimization of leukocyte concentration in platelet-rich plasma for the treatment of tendinopathy. J Bone Joint Surg Am 94: e143 (141–148).. 23032594 10.2106/JBJS.L.00019

[50] Djouad F, Mrugala D, Noel D, Jorgensen C (2006) Engineered mesenchymal stem cells for cartilage repair. Regen Med 1: 529–537. 17465847 10.2217/17460751.1.4.529

[51] Gaissmaier C, Fritz J, Krackhardt T, Flesch I, Aicher WK, et al (2005) Effect of human platelet supernatant on proliferation and matrix synthesis of human articular chondrocytes in monolayer and three-dimensional alginate cultures. Biomaterials 26: 1953–1960. 15576169 10.1016/j.biomaterials.2004.06.031

[52] Zaky SH, Ottonello A, Strada P, Cancedda R, Mastrogiacomo M (2008) Platelet lysate favours in vitro expansion of human bone marrow stromal cells for bone and cartilage engineering. J Tissue Eng Regen Med 2: 472–481. 18932128 10.1002/term.119

[53] Pittenger MF, Mackay AM, Beck SC, Jaiswal RK, Douglas R, et al (1999) Multilineage potential of adult human mesenchymal stem cells. Science 284: 143–147. 10102814 10.1126/science.284.5411.143

[54] Martin DR, Cox NR, Hathcock TL, Niemeyer GP, Baker HJ (2002) Isolation and characterization of multipotential mesenchymal stem cells from feline bone marrow. Exp Hematol 30: 879–886. 12160839 10.1016/s0301-472x(02)00864-0

[55] Hernigou P, Poignard A, Beaujean F, Rouard H (2005) Percutaneous autologous bone-marrow grafting for nonunions. Influence of the number and concentration of progenitor cells. J Bone Joint Surg Am 87: 1430–1437. 15995108 10.2106/JBJS.D.02215

[56] Veronesi F, Giavaresi G, Tschon M, Borsari V, Nicoli Aldini N, et al (2013) Clinical use of bone marrow, bone marrow concentrate and expanded bone marrow mesenchymal stem cells in cartilage disease. Stem Cells Dev 22: 181–192. 23030230 10.1089/scd.2012.0373

[57] Hermann PC, Huber SL, Herrler T, von Hesler C, Andrassy J, et al (2008) Concentration of bone marrow total nucleated cells by a point-of-care device provides a high yield and preserves their functional activity. Cell Transplant 16: 1059–1069. 18351022

[58] Weibrich G, Kleis WK, Hafner G, Hitzler WE, Wagner W (2003) Comparison of platelet, leukocyte, and growth factor levels in point-of-care platelet-enriched plasma, prepared using a modified Curasan kit, with preparations received from a local blood bank. Clin Oral Implants Res 14: 357–362. 12755786 10.1034/j.1600-0501.2003.00810.x

